# Supplementary material for: ATP-driven membrane binding and polymerization of bacterial actin MreB promotes local membrane fluidization
Source: Biophys J. 2026 Jun 3;125(14):3580–92. doi: 10.1016/j.bpj.2026.06.003 (PMC13507481; doi:10.1016/j.bpj.2026.06.003)
Supplement: Supplementary file 1 [file mmc1.pdf]

Biophysical Journal, Volume *125*

Supplemental information

ATP-driven membrane binding and polymerization of bacterial actin MreB promotes local membrane fluidization

Ingrid E. Adriaans, Ana Álvarez-Mena, Céline Dinet, Estelle Morvan, Kee Siang Lim, Erick J. Dufourc, Richard Wong, Arnaud Chastanet, Alphée Michelot, Rut Carballido-López, and Birgit Habenstein

ATP-driven membrane binding and polymerization of bacterial actin MreB promotes local membrane fluidization

Authors

Ingrid E. Adriaans[1#], Ana Álvarez-Mena[2#], Céline Dinet[3$], Estelle Morvan[4], Kee Siang Lim[5], Erick J. Dufourc[2], Richard Wong[5], Arnaud Chastanet[1], Alphée Michelot[3,6], Rut Carballido-López[1]* and Birgit Habenstein[2]*

Supplemental information

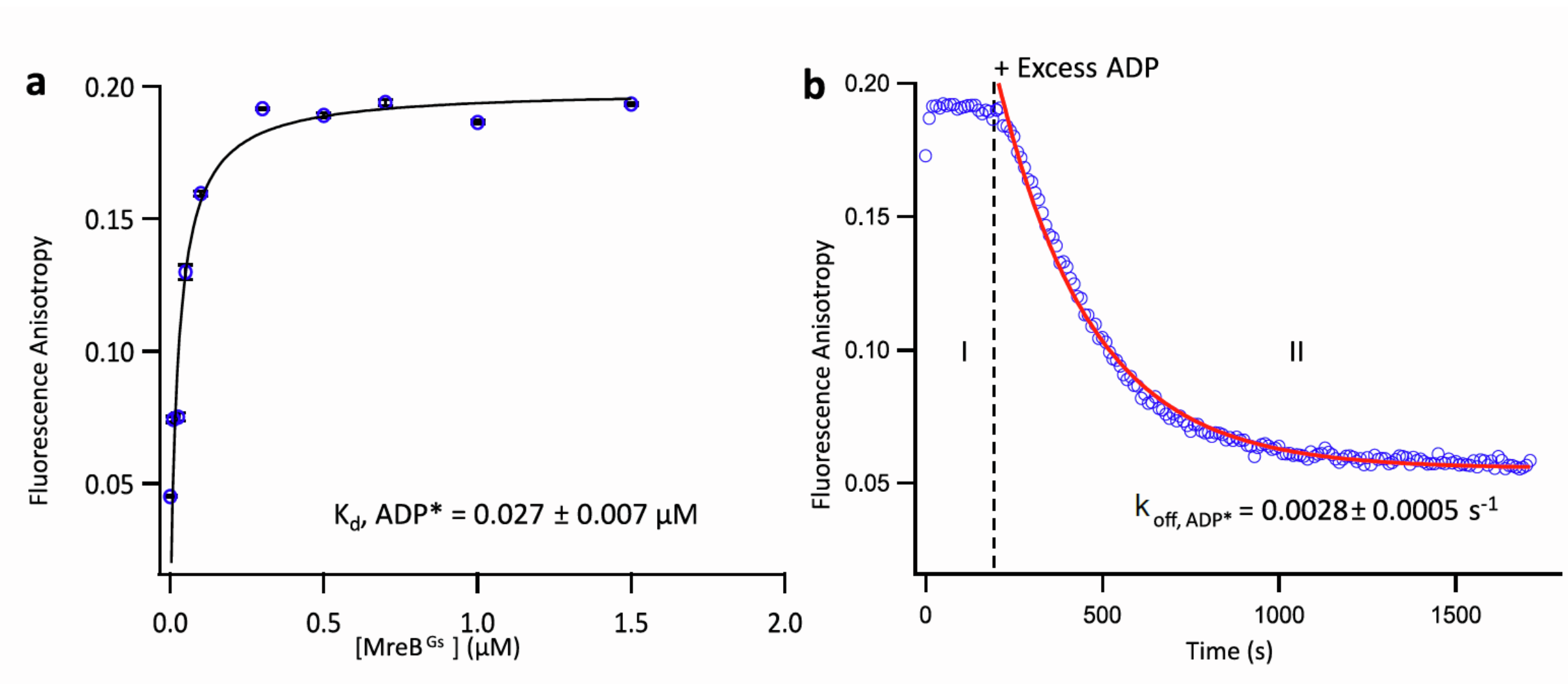

Supplementary Figure 1. Analysis of MreB^{Gs}–ADP* binding by fluorescence anisotropy. A. Fluorescence anisotropy measurements of N6-(6-amino)hexyl-ADP-ATTO-488 (0.2 µM - referred to as ADP*) to Mg-MreB^{Gs} (1 µM). Binding was assessed by titrating increasing concentrations of MreB^{Gs} against a fixed concentration of ADP* (0.2 µM), and measuring fluorescence anisotropy at steady state (5 minutes after mixing). Dissociation constants (K_d) were determined by fitting the binding curves. Each data point represents the mean ± standard deviation from at least three independent measurements. **B.** Binding kinetics of ADP* (0.2 µM) to MreB^{Gs} (1 µM), followed in real time by fluorescence anisotropy. At 230 seconds, excess unlabeled ADP (1 mM) was added (dashed line), displacing bound ADP*. Red line: fitting of the dissociation phase (II) yields the $k_{off,ATP*}$ value for ADP* release.

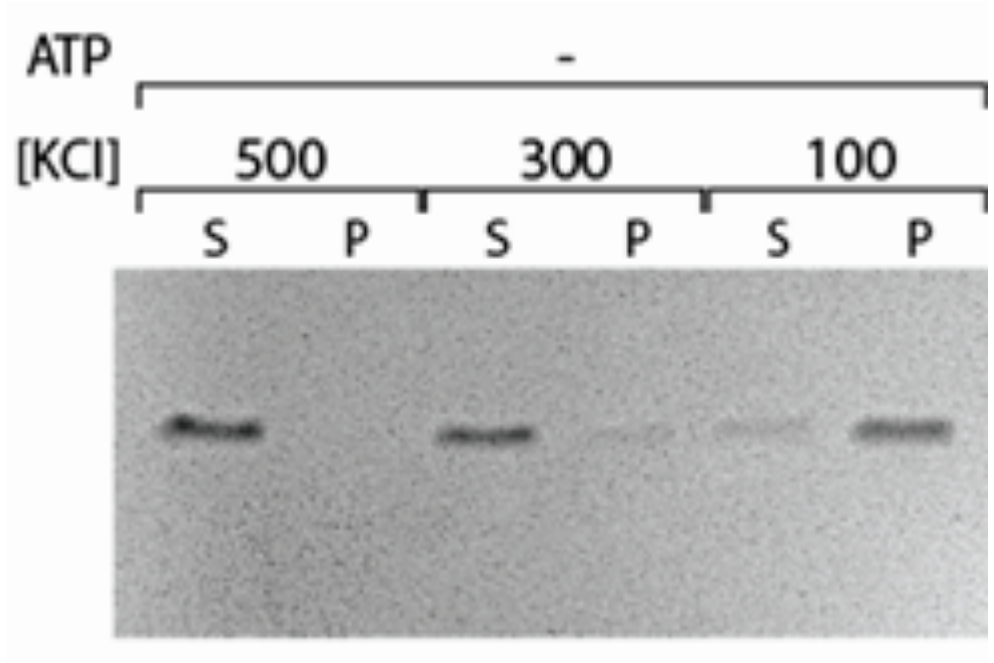

Supplementary Figure 2. The Ionic strength affects MreB^{Gs} binding to lipids in a liposome pelleting assay. 12% SDS-PAGE showing sedimentation of MreB^{Gs} (1.3 µM) in the presence of *E. coli* lipid polar extract liposomes (0.5 mg/ml) in the absence (-) of ATP. The reactions contain either 100, 300 or 500 mM KCl. S, supernatant; P, pellet.

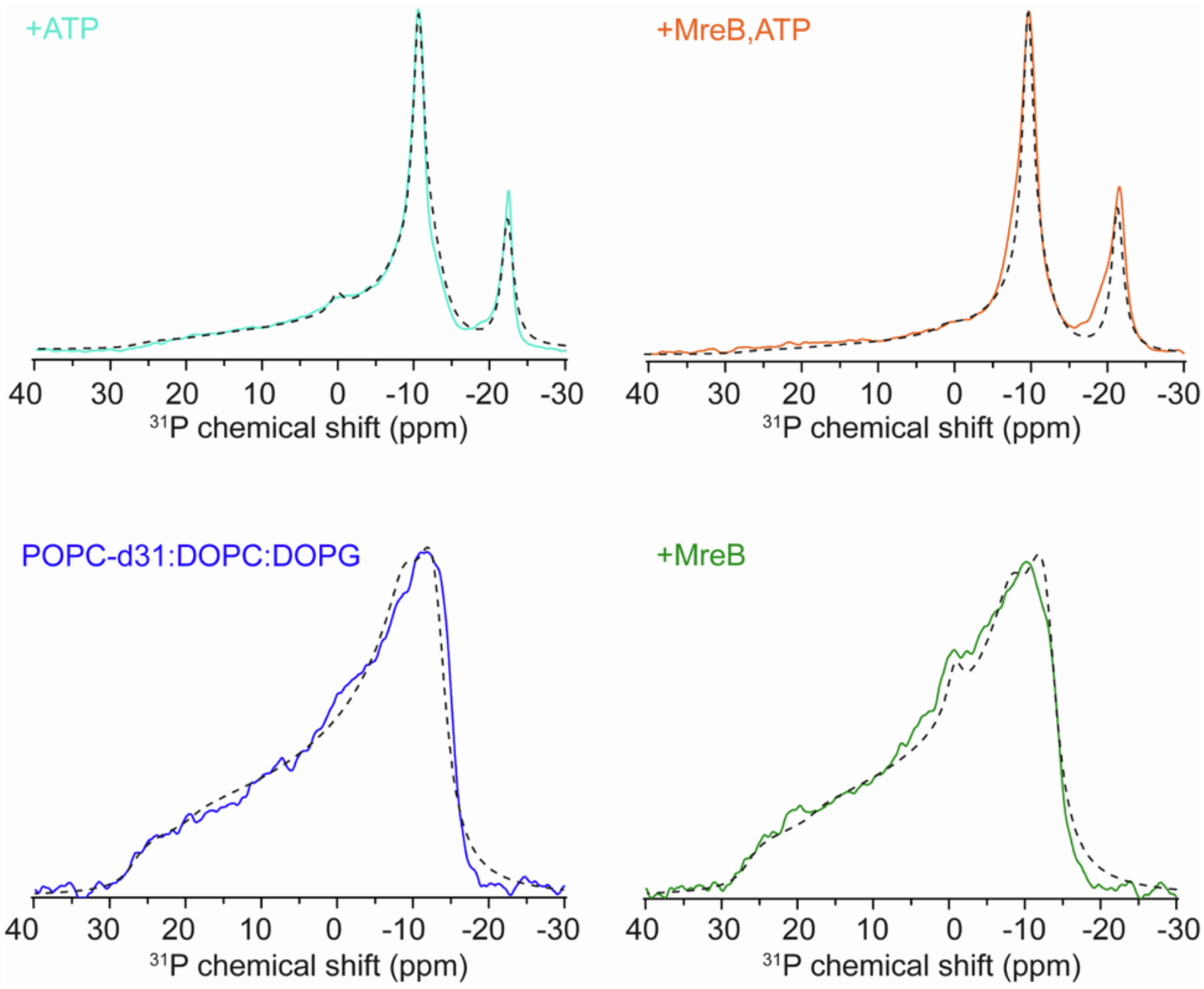

Supplementary Figure 3. Simulated ^{31}P NMR spectra (black dashed lines) of POPC-d31:DOPC:DOPG (35:45:20) at 303 K under different conditions, overlaid on recorded spectra (plain colored lines): buffer alone (blue), ATP alone (cyan), MreB^{Gs} alone (green) and both MreB^{Gs} and ATP (orange).

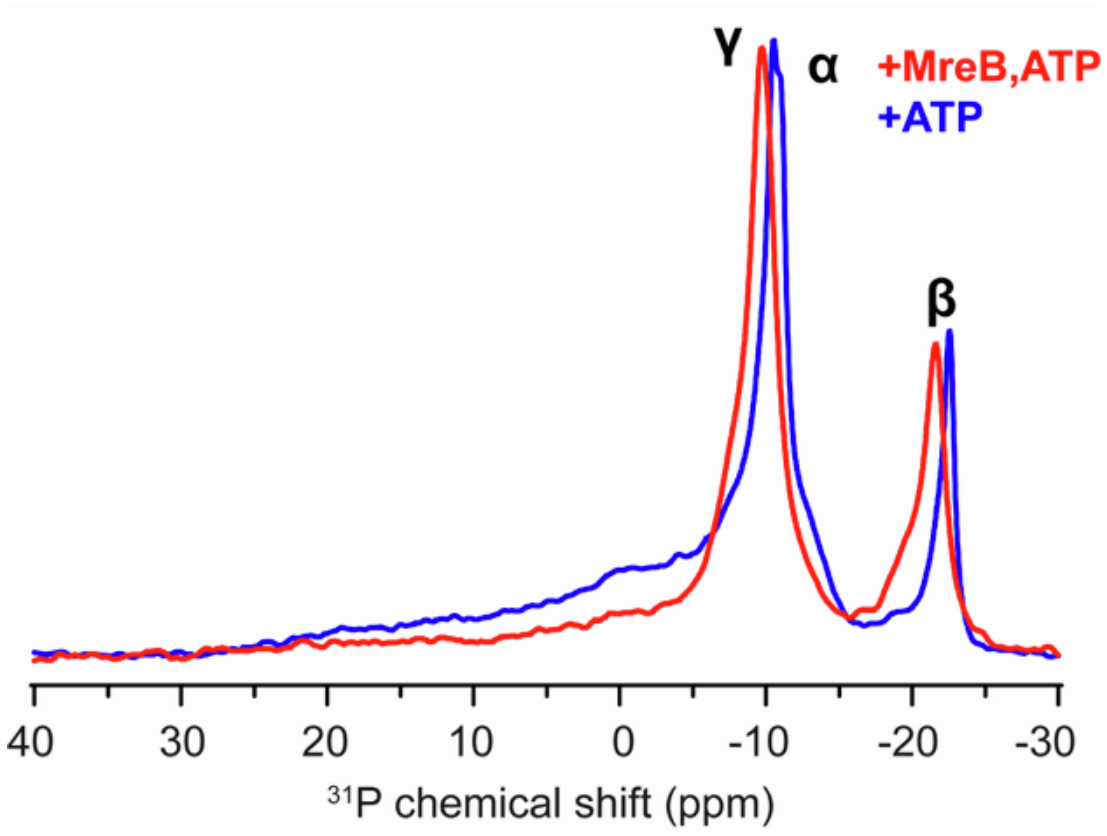

Supplementary Figure 4. Superimposition of ^{31}P NMR spectra of POPC-d31:DOPC:DOPG multilamellar vesicles (35:45:20) recorded at 303 K in the presence of ATP (blue) and MreB + ATP (red), showing the assignments of the three phosphate groups of ATP.

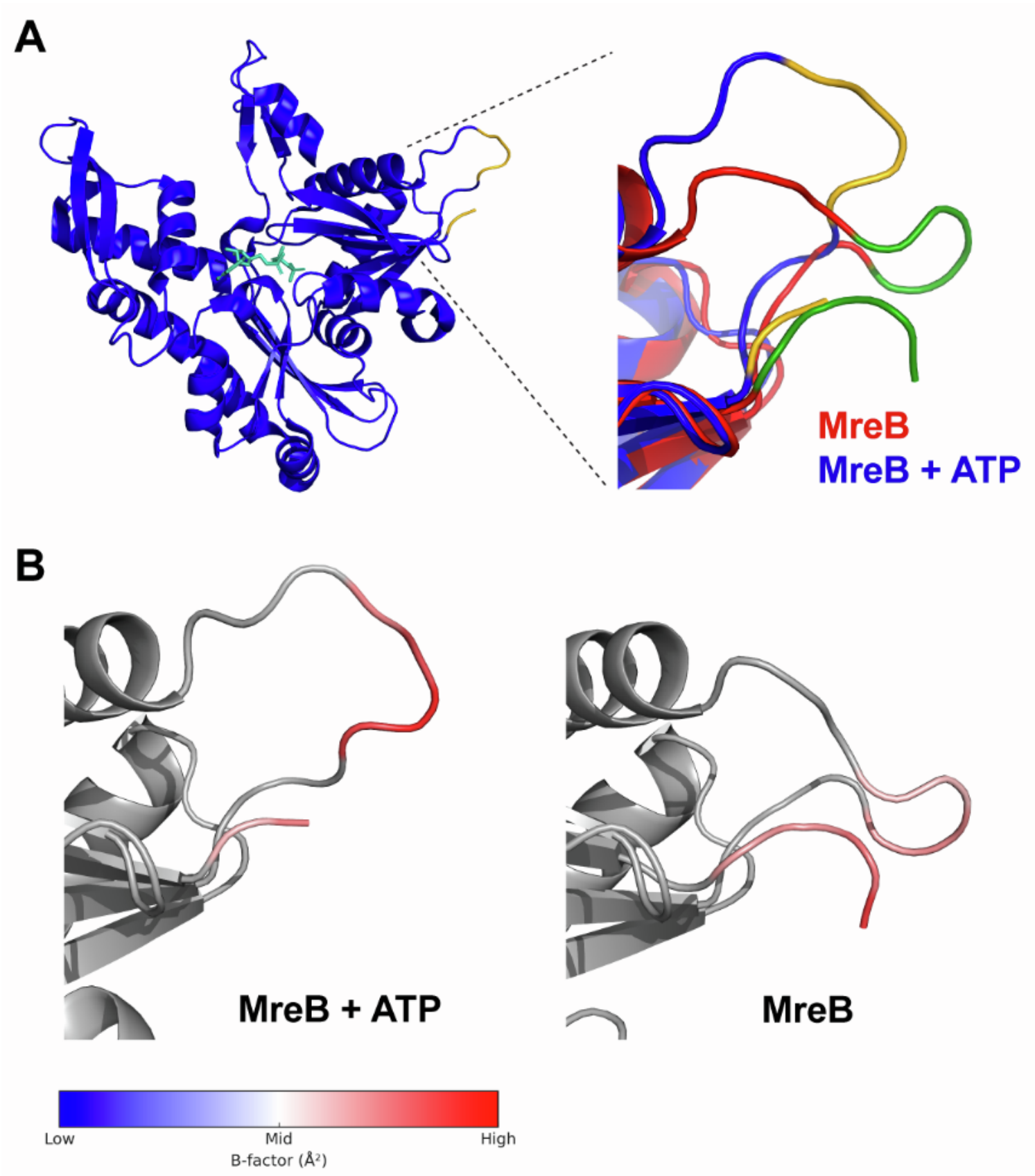

Supplementary Figure 5. Local flexibility in the membrane-binding region of apo and ATP-bound MreB. A. Left: Crystal structure of ATP-bound MreB from *G. stearothermophilus* (PDB ID 8AZG), highlighting the partial N-terminal region and the α2-β7 loop (yellow), which are essential for membrane binding and polymerization on lipid surfaces. Right: zoom in and structural overlay of apo $MreB^{Gs}$ (PDB ID: 7ZPT, red, with the N-terminal region and α2-β7 loop highlighted in green) and ATP-bound $MreB^{Gs}$ (PDB ID: 8AZG, blue) within the membrane-binding region, showing a conformational shift associated with nucleotide binding. **B.** Zoom up representation of apo MreB (PDB ID: 7ZPT) and ATP-bound MreB (PDB ID: 8AZG), showing the N-terminal segment and the α2-β7 loop, which are highlighted and colored according to their B-factor values, which were extracted from the PDB files using PyMOL and averaged per residue. Higher B-factors reflect greater atomic mobility and flexibility, highlighting regions with increased dynamic behavior upon nucleotide binding.

Supplementary Table 1: Simulated quadrupolar splittings $\Delta \nu_Q$ of the POPC-d31 acyl chain in POPC-d31:DOPC: DOPG (35:45:20, w/w) vesicles under the four experimental conditions.

Deuterated carbon position	Experimental condition			
	Buffer	Buffer + ATP	Buffer + MreB	Buffer + MreB + ATP
	$\Delta \nu_Q$	$\Delta \nu_Q$	$\Delta \nu_Q$	$\Delta \nu_Q$
**2**	51	50	52.5	51
**3**	51	50	52.5	51
**4**	51	50	50	51
**5**	47.5	50	48	48.8
**6**	47	47	47	45
**7**	43.8	46.5	45.5	42
**8**	42.8	43	42.5	39.5
**9**	39.8	40	40	35.5
**10**	34.2	34.3	34.5	30
**11**	30	30.5	30	25
**12**	25	25.5	25	19
**13**	21	21	20.5	15
**14**	15.2	15.3	15	8.7
**15**	6	8	6	4.3
**16**	4	4	4	2

Supplementary Table 2: ATP ^{31}P chemical shifts at positions α, β and γ.

Sample	^{31}P chemical shift ATP (ppm)		
	α	β	γ
**ATP**	-11.1	-22.6	-10.5
**MreB + ATP**	-10.1	-21.7	-9.7
